# Refractory HIV-Associated Guillain–Barré Syndrome Responsive to Antiretroviral Therapy: A Case Report

**DOI:** 10.1155/crdi/7292001

**Published:** 2024-11-20

**Authors:** Sean Coyle, Ian Sutherland Cormack

**Affiliations:** Heath Clinic, Croydon University Hospital, Croydon, London, UK

**Keywords:** antiretroviral, Guillain–Barré syndrome, HIV, peripheral neuropathy

## Abstract

Guillain–Barré Syndrome (GBS) is an acute polyneuropathy commonly preceded by infection, with growing recognition of the human immunodeficiency virus (HIV) as a trigger. We present a case of a 44-year-old male with HIV-associated GBS refractory to intravenous immunoglobulin (IVIG) therapy, who achieved remission upon starting highly active antireroviral therapy (HAART). There remains a lack of consensus on the management of this condition across the spectrum of disease, and the interplay between the therapeutic options is poorly understood. This report aims to add to the current body of knowledge on this rare condition and highlight the need for retrospective analysis of the currently available literature.

## 1. Introduction

GBS is a rare but well-recognised immune-mediated demyelinating polyneuropathy caused by autoimmune destruction of peripheral nerves. The pathophysiology is widely accepted to be a mechanism of molecular mimicry and immune cross-reaction, with approximately 75% of cases featuring an antecedent infection [1, 2]. Around one third of cases are attributable to Campylobacter jejuni, while *Mycoplasma pneumoniae* and Cytomegalovirus (CMV) can also be implicated [1]. GBS secondary to HIV infection is less well recognised but has been increasingly identified since the epidemic of the 1980s, with case reports forming the majority of the literature [3–9]. Indeed, GBS is currently regarded as a clinical indicator condition for HIV testing, whereby failure to test in this context represents a missed opportunity [10]. There is currently limited data supporting specific management approaches in relation to clinical outcome. This report aims to broaden the current data pool by outlining a case of refractory HIV-associated GBS which achieved good clinical outcome following initiation of HAART.

## 2. Case

A previously fit and well 44-year-old male presented to hospital with a 4-day history of bilateral paraesthesia in the hands and feet, which had developed into lower back and bilateral leg pain. Neurological examination demonstrated paraesthesia and patchy weakness in all four limbs, with sensory deficits at the L5-S2 dermatomes and variable loss of power ranging from one to four out of five. Initial blood tests and MRI imaging of the spinal column were unremarkable. Clinically, the symptoms of paraesthesia and weakness worsened with proximal advancement and new oropharyngeal dysphagia.

Lumbar puncture with CSF analysis revealed a white cell count of 17/*μ*L and protein of 1.82 g/L, but no microorganisms on microscopy nor growth on culture.

Nerve conduction studies confirmed a moderate to severe generalised demyelinating sensorimotor polyneuropathy. Specific findings included prolonged or absent F-waves, slowing of motor conduction and prolonged distal motor latencies with sural-sparing sensory involvement. No evidence of axonal loss was demonstrated in this study.

The patient was commenced on IVIG (Octagam 30 g OD) for 5 days with no improvement in existing symptoms, subsequently developing new diplopia and eyelid dysfunction. As per trust policy for HIV opt-out testing on all admissions, this patient's blood had been screened and found to be reactive. Results were positive in three subsequent confirmatory HIV assays. Further testing confirmed a HIV viral load of 2,260,000 copies and a CD4 count of 240 cells/μL. The patient was commenced on HAART with Truvada (TDF/FTC) + Dolutegravir.

By the third day of HAART, the viral load had dropped to 152,000 copies, which was associated with an abatement of oropharyngeal dysphagia. Campylobacter serological testing was unavailable, but screens for other common antecedent infections were negative, implicating HIV. Incidentally, the patient also had reactive syphilis serology but had begun to improve with HAART before syphilis treatment was initiated, suggesting this diagnosis was not relevant to the presentation.

At completion of 1 week HAART, there was clinical improvement generally in motor function, although lower limb weakness and balance difficulties greatly limited mobility. After inpatient rehabilitation, the patient remained partially reliant on a wheelchair and was transferred into a programme of neuro-rehabilitation in the community.

Upon discharge from community services 1 year later, the patient had achieved significant functional improvement; namely, independent ambulation, the ability to stand up from the floor without assistance, as well as improvement in balance, manual dexterity and grip strength. The patient continues to live independently and at the time of writing remains engaged with HIV care.

## 3. Discussion

The growing recognition of HIV as a precipitant of GBS is reflected in scatterings of case reports, with occasional case series and literature reviews. Much of the literature reflects a trial-and-error approach to management, with variable success across the spectrum of HIV chronicity and ART exposure. There remains little definitive or comparative data on treatment success rates, and how this varies according to antiretroviral naivety, choice of antiretroviral therapy or the stage of HIV disease at GBS onset.

The existing body of the literature indicates acute HIV infection, or seroconversion, as the most frequent point of GBS onset [8]. However, GBS has also been observed across the spectrum of HIV disease, at chronic stages, in both treated and untreated patients, or at the point of immune reconstitution [4–12]. This is further complicated by the capacity of HIV itself to manifest as various forms of peripheral neuropathy [3, 13]. In addition, in advanced disease with significantly reduced immune reserve, the presence of opportunistic pathogens such as CMV or Varicella Zoster Virus (VZV) can act as a precipitant for GBS, making true cases more challenging to discern [12].

The mainstay of GBS management is IVIG or plasmapheresis, with HAART being added to the therapeutic arsenal in the specific context of HIV-associated GBS. The almost immediate abatement of symptoms in this case would indicate the benefit of HAART, particularly as first-line GBS therapies had been unsuccessful. The interplay between these therapies is yet to be fully elucidated, and the body of currently available case reports demonstrates variable or incongruous results. While the timing of symptom dissipation in this case seems to be the direct result of HAART, it does not exclude the possibility of two therapies working synergistically. Indeed, one retrospective analysis found that IVIG with initiation of ART conferred greater functional outcome than initiating ART alone [14]. Conversely, there are case reports of patients who responded to standard GBS therapies prior to the initiation of HAART [8]. This area, with its conflicting reports, would likely benefit from a comparative retrospective analysis and literature review of the current case volume.

## 4. Conclusion

There are growing numbers of case reports illustrating GBS in response to HIV infection. There is a need for greater recognition of HIV as a precipitating factor for GBS with emphasis on more thorough testing. HAART may play an important role in management of HIV-associated GBS as an indirect agent that works to eliminate the precipitating infection, but its interaction and synergy with the other mainstay treatments of GBS is not well understood. Due to the rarity and short-lived nature of the condition, there are limited opportunities for case-control research, but this subject matter may lend itself to a narrative synthesis style analysis of the currently available case reports in order to identify any emerging trends with clinical implications.

## Data Availability

All data generated or analysed during this study are included within this article. Further enquiries can be directed to the corresponding author.
